# Differential relationships between apathy and depression with white matter microstructural changes and functional outcomes

**DOI:** 10.1093/brain/awv304

**Published:** 2015-10-21

**Authors:** Matthew J. Hollocks, Andrew J. Lawrence, Rebecca L. Brookes, Thomas R. Barrick, Robin G. Morris, Masud Husain, Hugh S. Markus

**Affiliations:** 1 Stroke Research Group, University of Cambridge, Department of Clinical Neurosciences, R3, Box 183, Addenbrooke’s Biomedical Campus, Cambridge, CB2 0QQ, UK; 2 St. Georges, University of London, Neurosciences Research Centre, Cardiovascular and Cell Sciences Research Institute, London, UK; 3 King’s College London, Institute of Psychiatry, Psychology and Neuroscience, Department of Psychology, London, UK; 4 University of Oxford, Nuffield Department of Clinical Neurosciences, Oxford, UK

**Keywords:** diffusion tensor imaging, emotion, lacunar stroke, motivation, vascular dementia

## Abstract

Small vessel disease is a stroke subtype characterized by pathology of the small perforating arteries, which supply the sub-cortical structures of the brain. Small vessel disease is associated with high rates of apathy and depression, thought to be caused by a disruption of white matter cortical-subcortical pathways important for emotion regulation. It provides an important biological model to investigate mechanisms underlying these key neuropsychiatric disorders. This study investigated whether apathy and depression can be distinguished in small vessel disease both in terms of their relative relationship with white matter microstructure, and secondly whether they can independently predict functional outcomes. Participants with small vessel disease (*n* = 118; mean age = 68.9 years; 65% male) defined as a clinical and magnetic resonance imaging confirmed lacunar stroke with radiological leukoaraiosis were recruited and completed cognitive testing, measures of apathy, depression, quality of life and diffusion tensor imaging. Healthy controls (*n* = 398; mean age = 64.3 years; 52% male) were also studied in order to interpret the degree of apathy and depression found within the small vessel disease group. Firstly, a multilevel structural equation modelling approach was used to identify: (i) the relationships between median fractional anisotropy and apathy, depression and cognitive impairment; and (ii) if apathy and depression make independent contributions to quality of life in patients with small vessel disease. Secondly, we applied a whole-brain voxel-based analysis to investigate which regions of white matter were associated with apathy and depression, controlling for age, gender and cognitive functioning. Structural equation modelling results indicated both apathy (r = −0.23, *P* ≤ 0.001) and depression (r = −0.41, *P* ≤ 0.001) were independent predictors of quality of life. A reduced median fractional anisotropy was significantly associated with apathy (r = −0.38, *P* ≤ 0.001), but not depression (r = −0.16, *P* = 0.09). On voxel-based analysis, apathy was associated with widespread reduction in white matter integrity, with the strongest effects in limbic association tracts such as the anterior cingulum, fornix and uncinate fasciculus. In contrast, when controlling for apathy, we found no significant relationship between our white matter parameters and symptoms of depression. In conclusion, white matter microstructural changes in small vessel disease are associated with apathy but not directly with depressive symptoms. These results suggest that apathy, but not depression, in small vessel disease is related to damage to cortical-subcortical networks associated with emotion regulation, reward and goal-directed behaviour.

## Abstract (precis)

Small vessel disease pathways disrupts subcortical pathways that are important for emotion regulation. Hollocks *et al.* use brain imaging and statistical modelling to show that white-matter damage is associated with apathy, but not depression, although the latter still has a significant impact on quality of life.

## Graphical abstract

**Figure:**
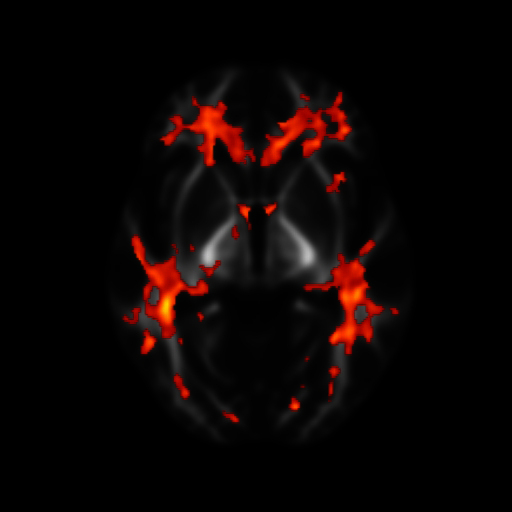
Small vessel disease pathways disrupts subcortical pathways that are important for emotion regulation. Hollocks *et al.* use brain imaging and statistical modelling to show that white-matter damage is associated with apathy, but not depression, although the latter still has a significant impact on quality of life.

## Introduction

People with cerebrovascular disease often present with neuropsychiatric symptoms, with depression and apathy being particularly prevalent, occurring in ∼30% of all stroke (Hackett *et al.*, 2014). Depressive symptoms have been particularly associated with the cerebral small vessel disease (SVD) subtype of stroke (Brookes *et al.*, 2014*a*). SVD is characterized by pathology of the small perforating arteries, which supply the deep white matter and grey matter structures of the brain, leading to white matter lesions and lacunar infarcts, with ∼40% of patients with lacunar stroke showing cognitive impairment (Makin *et al.*, 2013). The pathology is predominantly subcortical, although recent data have suggested microinfarcts may also occur in the cortical grey matter (Smith *et al.*, 2012).

It has been suggested that the increased prevalence of depression in SVD (White *et al.*, 2011) may be due in part to the disruption of white matter pathways underlying subcortical-cortical networks involved in mood regulation (Brookes *et al.*, 2014*a*), a hypothesis supported by an association between MRI white matter hyperintensities and late-life depression (O’Brien *et al.*, 2006; Sneed *et al.*, 2008; Tang *et al.*, 2010). Such symptoms are often undetected, and have a significant impact on quality of life (Brookes *et al.*, 2013). There is much less work on apathy in SVD, although it has been suggested that it is particularly associated with this stroke subtype (Moretti *et al.*, 2015), and is a common symptom in dementia patients with coexistent white matter changes (Hahn *et al.*, 2013).

A challenge in describing apathy in SVD is the considerable overlap with symptoms of depression, and the resultant difficulty in differentiating the two clinically (Landes *et al.*, 2005). Apathy is defined as a loss of, or diminished, motivation, in combination with reduction in either goal-directed behaviour, cognitive activity or emotional expression (Marin *et al.*, 1991; Robert *et al.*, 2009). However, reduced motivation is also an important factor in depression, and anhedonia, or a loss of interest or pleasure, and is one of nine primary symptoms of major depressive disorder in the Diagnostic and Statistical Manual of Mental Disorders, Fifth Edition (DSM-V) (Uher *et al.*, 2013). Despite this substantial overlap, there is considerable evidence that apathy and depression are distinguishable and dissociable from each other (Marin *et al.*, 1993; Starkstein *et al.*, 2005; Levy and Czernecki, 2006; van der Mast *et al.*, 2008; Kirsch-Darrow *et al.*, 2011). It has been proposed that apathy is caused by impairments in decision-making, related to disruption of connections between the prefrontal cortex and the basal ganglia and other subcortical structures, which prevent a person from integrating ‘emotional-affective signals’ with the selection of future behaviour (Levy and Dubois, 2006).

Diffusion tensor imaging (DTI) is a non-invasive technique used to evaluate the structural integrity of white matter in the brain, and has been shown to be sensitive to white matter damage in SVD (van Norden *et al.*, 2012; Lawrence *et al.*, 2013). DTI studies investigating white matter changes associated with apathy and depression in a variety of neurodegenerative conditions have demonstrated considerable overlap between the two (Apostolova *et al.*, 2007; Cacciari *et al.*, 2010; Shackman *et al.*, 2011; Hahn *et al.*, 2013; Kim *et al.*, 2013; Spalletta *et al.*, 2013; Stanton *et al.*, 2013). However, the interpretation of these studies is challenging because apathy and depression are rarely studied together in a single sample. To date there have been no studies examining the white matter correlates of apathy in people with SVD.

A recent meta-analysis of DTI studies identified a number of white matter tracts associated with late-life depression (Wen *et al.*, 2014). Depression in SVD has previously been associated with white matter damage (Brookes *et al.*, 2014*a*), as well as regional reductions in fractional anisotropy in the genu and body of the corpus callosum, the right anterior cingulum and the bilateral inferior fronto-occipital fasciculus, uncinate fasciculus and corona radiata (van Uden *et al.*, 2015). However, it is currently unknown whether any of these regions are related to apathy in SVD. In this study we determined the prevalence of, and overlap between, apathy and depression in patients with SVD, using indices that have been developed in community-based studies of ageing. We analysed the results using structural equation modelling, which allows the concurrent modelling of multiple linear associations testing both overall model fit and individual path strength. The relationships between white matter microstructure, apathy and depression were determined, while also quantifying their relative contributions to quality of life, taking into account the impact of any cognitive impairment. Finally, to provide insights into the potential mechanisms of neuropsychiatric comorbidities in SVD, we examined which regions of white matter microstructure were specifically related to either apathy or depression.

## Materials and methods

### Participants

Patients with symptomatic SVD (*n* = 121) were recruited to the prospective longitudinal St. George’s Cognition and Neuroimaging in Stroke (SCANS) study (Lawrence *et al.*, 2013). This study includes data from baseline only. One participant was excluded from the primary analysis due to having missing questionnaire and cognitive data and a further two participants were excluded from the voxel-based analysis as they were unable to complete the MRI protocol. SVD was defined as a clinical lacunar stroke syndrome associated with an anatomically appropriate lacunar infarct on MRI, with additional confluent leukoariosis rated as Fazekas grade 2 or higher (Fazekas *et al.*, 1987). Exclusion criteria included any type of stroke other than SVD, including extra- or intracranial large artery stenosis (≥50%), a cardio-embolic source, non-lacunar subcortical infarcts which are >1.5 mm in diameter, cortical infarcts or a history of any other neurological or psychiatric disorder, with the exception of depression. The study was reviewed and approved by the Wandsworth research ethics committee. As a normal comparison we used data from 398 healthy controls recruited as part of the Brief Memory and Executive Test multi-site validation study (Brookes *et al.*, 2012, 2015), but with overlapping questionnaire and background measures (Table 1). Controls were recruited from local family doctors’ practices or other volunteer groups. Individuals with cardiovascular risk factors and other comorbidities were included, but individuals with a past history of stroke, transient ischaemic attack, major central neurological or psychiatric disease were excluded. All patient assessments were performed at least 3 months post-stroke to reduce effects of acute ischaemia on cognition.

**Table 1:** Descriptive statistics of demographic and clinical variables for participants with SVD and controls

**Measure** (mean, SD)	**Control** (*n* = 398)	**SVD** (*n* = 118)	***t*-test**
Age (years)	61.9 (13.5)	69.9 (9.8)	*t* = 2.6 (*P* ≤ 0.01)
Gender (% male)	52	65	-
Geriatric Depression Scale – Apathy	1.5 (1.5)	2.7 (1.3)	*t* = 8.8 (*P* ≤ 0.01)
Geriatric Depression Scale – Depression	3.6 (3.4)	10.7 (3.1)	*t* = 6.4 (*P* ≤ 0.01)
Weight (kg)	74.0 (23.1)	76.5 (15.9)	*t* = .86 (*P* = 0.37)
Systolic blood pressure (mmHg)	131.9 (17.2)	146.8 (21.5)	*t* = 7.6 (*P* ≤ 0.01)
Diastolic blood pressure (mmHg)	79.4 (10.1)	80.9 (10.8)	*t* = 3.9 (*P* = 0.18)
Hypertension (%)	73	93	-
Hypercholesterolaemia (%)	73	88	-
Diabetes mellitus (%)	7	20	-
Current smoker (%)	10	20	-
Cognitive scores*			
Global cognitive function	-	−0.58 (0.85)	-
Processing speed	-	−0.73 (0.93)	-
Executive functioning	-	−0.86 (1.1)	-
Memory	-	−0.06 (0.99)	-

Hypertension and hypercholesterolaemia are % being treated; *Cognitive scores are presented as mean *z*-scores.

#### Questionnaire measures of apathy, depression and quality of life

The Geriatric Depression Scale was used to measure depression. The Geriatric Depression Scale is a 30-item self-report screening scale for depression that asks participants to answer ‘yes’ or ‘no’ to questions concerning depression symptoms. The scale has good internal consistency (α = 0.86) (Yesavage *et al.*, 1983), and a sensitivity of ∼80% when compared to clinical diagnoses of depression (Mitchell *et al.*, 2010). An ‘apathy scale’ was created using six of the Geriatric Depression Scale items (Adams *et al.*, 2004), and consisted of the following items: ‘prefer to stay at home’, ‘avoid social gatherings’, ‘dropped activities and interests’, ‘find life very exciting’, ‘hard to start new projects’ and ‘full of energy’. The depression scale did not include the six apathy items and had a range of 0–24, while the apathy scale had a range of 0–6. To confirm the use of these scales in our sample, a confirmatory factor analysis was conducted (see below).

The Stroke Specific Quality of Life Scale (SS-QoL) was developed specifically to measure quality of life in stroke trials, measuring multiple domains of quality of life as well as providing a global score (Williams *et al.*, 1999). Because the SS-QoL was developed for a single post-acute stroke assessment, the scale items were modified into the present tense to reflect general current experiences (e.g. ‘I often have to stop and rest during the day’). The SS-QoL has a specific domain for mood problems, which for the purposes of this study was not included in the total score, to minimize confounding effects with the depression measure.

#### Neuropsychological measures

Assessment was performed by a neuropsychologist using a battery of widely-used tasks chosen to characterize the cognitive impairment seen in SVD, and taking ∼2.5 h to administer. Following a previously used methodology (Lawrence *et al.*, 2013), a series of cognitive index scores were created to summarize performance across tasks for executive functioning, processing speed and long-term memory, while a global cognitive index score was produced which summarized performance across all tasks. For a list of the neuropsychological tasks included in the global cognitive score and each index score (Supplementary material). To construct index scores, the primary measures for each task were transformed into *z*-scores using age-scaled normative data. These scores were aggregated to form the indices by averaging the resulting measures showing adequate internal consistency (executive functioning α = 0.75, processing speed α = 0.65, long-term memory a = 0.73, global index score α = 0.94) (Lawrence *et al.*, 2013).

### Diffusion tensor imaging

Images were acquired from a 1.5 T General Electric Signa HDxt (General Electric), maximum gradient amplitude 33 mTm^−1^, using a proprietary head coil. All sequences had whole brain coverage and the total acquisition time was 45 min. Axial single shot spin echo planar images were acquired (repetition time/echo time = 15 600/93.4 ms, field of view = 240 × 240 mm^2^, matrix = 96 × 96, 55 slices of 2.5 mm with no slice gap). Eight volumes without diffusion weighting (b = 0 smm^−2^) were followed by 25 diffusion-sensitized images with gradients applied in non-collinear directions (b = 1000 smm^2^). These acquisitions were repeated to obtain four more unweighted volumes and the negative of the 25 gradients.

Images were realigned to remove eddy current distortions using FMRIB’s Linear Image Registration Tool (FLIRT) (Jenkinson and Smith, 2001), part of the FMRIB Software Library (FSL) (Smith *et al.*, 2004; Jenkinson *et al.*, 2012), version 5.0. Slices with signal loss due to motion were identified using an in-house intensity-based program and excluded from further analysis. Most diffusion tensor images (111/118) were identified as requiring some degree of correction by excluding diffusion-weighted image slices with excessive patient movement prior to computation of the DTI. In most cases slice exclusion was minimal (median 8.5/2750 slices, interquartile range = 14.75 slices). The maximum number of excluded slices was 133 over 50 diffusion-weighted image volumes (i.e. there are 55 slices per diffusion-weighted gradient direction image meaning that ∼4.83% of slices for that individual were excluded). All corrected and uncorrected diffusion tensor images were visually inspected prior to further analysis. Diffusion-weighted volumes with opposite gradients were geometrically averaged to eliminate gradient cross-terms (Neeman *et al.*, 1991). The eight b = 0 smm^−2^ images were co-registered and the average taken to provide a T_2_-weighted echo planar image, which we term the ‘b0’ image.

A mask which excluded non-brain tissue was created from the b0 image using FSL’s Brain Extraction Tool (Smith, 2002). Masks were inspected and manually corrected where necessary.

Diffusion tensors were fitted using the least squares method (Basser *et al.*, 1994) for each voxel of the brain mask by FSL’s ‘dtifit’ program. The tensor was decomposed to create maps of fractional anisotropy and mean diffusivity. When disease-related white matter changes are present you would expect to observe reduced fractional anisotropy, and increased mean diffusivity values. Prior to voxel-wise analysis the fractional anisotropy images underwent binary erosion with a 3 × 3 × 1 voxel kernel.

### Voxel-based analysis

Analysis of fractional anisotropy and mean diffusivity maps was conducted using a voxel-based analysis methodology described by Schwarz and colleagues (2014). This combines the use of an advanced registration technique implemented in the Advanced Normalization Tools (ANTS) software (Klein *et al.*, 2009; Avants *et al.*, 2011) with an iteratively generated unbiased group-template to allow valid voxel-wise comparisons (Schwarz *et al.*, 2014), similar to the voxel-based morphometry technique (VBM) (Ashburner and Friston, 2000).

To create a standard fractional anisotropy template with minimal bias for use in the voxel-based analysis we adopted an iterative procedure with three stages. Linear registrations were implemented in FSL FLIRT using default settings (12 parameter affine transformation computed using the correlation ratio cost function). First, a representative subject fractional anisotropy image was linearly registered to the FMRIB58 fractional anisotropy template provided with FSL. All images were linearly registered to this initial target and the mean average of these images used to create an initial group template. The template was updated with two further iterations of the above steps. The final mean average image was used to initialize a non-linear template generated using ANTS via the buildtemplateparallel.sh script (Avants *et al.*, 2011). As a final step, a linear transformation was computed between the non-linear group template and the FMRIB58 template. The above transformations bringing fractional anisotropy and mean diffusivity maps in subject space to the FMRIB58 template analysis space (via the group template) were concatenated and the combined transform applied in a single step to minimize interpolation errors. Following previously used methodology, the voxel-based analysis of all subject images were smoothed using a Gaussian kernel (σ = 1 mm) and analysis was restricted to voxels with an average fractional anisotropy ≥0.2 representing regions of mostly white matter (Schwarz *et al.*, 2014).

In order to include whole white matter summary measures of fractional anisotropy and mean diffusivity in the structural equation model (see below) we extracted the median fractional anisotropy and mean diffusivity for all non-zero white matter voxels included within the voxel-based analysis mask using the ‘fslstats’ function in FSL.

### Statistical analysis

We compared firstly the severity of apathy and depression between the SVD cases and controls and then thresholded the scale using median splits to identify high and low scoring participants, allowing us to establish the degree of overlap between apathy and depression in the group of patients with SVD. To confirm validity of the apathy subscale in our sample, we conducted a confirmatory factor analysis based on the six factor model (Adams *et al.*, 2004).

#### Structural equation model

Structural equation modelling is a statistical technique which enables the testing of a theoretical model that includes a set of directional (regression) and non-directional relationships (covariances). This allows the quantification of the independent contributions of predictor variables on outcome variables, while accounting for dependencies between them. Here we used multilevel structural equation modelling to investigate the relationship between white matter microstructure and apathy, depression, global cognitive impairment and the final outcome variable, quality of life; this was done within the SVD sample. However, we also wanted to identify any independent contributions of apathy, depression and global cognitive functioning on quality of life. Overall, this allows us to estimate the independent contributions of both apathy and depression on quality of life while taking into account relationships with white matter changes and any inter-relationships between the other observed variables, age and IQ included, within the covariation matrix.

**Figure 1:**
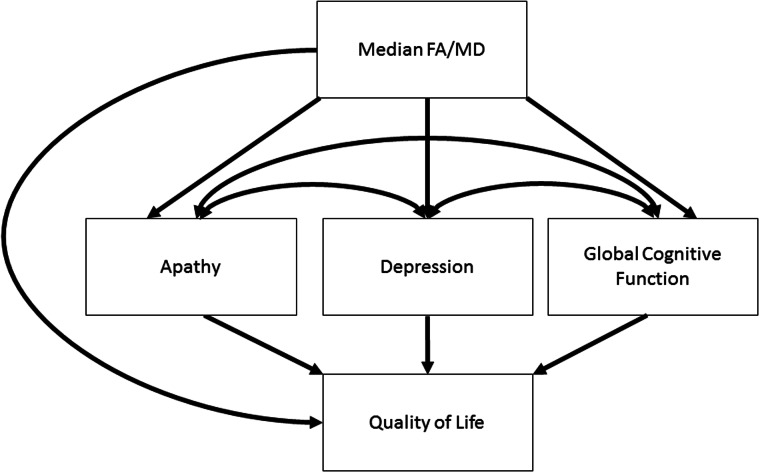
**Hypothesized model examining the relationship between median fractional anisotropy/mean diffusivity, apathy, depression, cognitive impairment and quality of life**. FA = fractional anisotropy; MD = median diffusivity. NART errors and age are regressed onto all independent variables.

For the structural equation modelling analysis, key variables and covariates were included in an initial hypothesized model (Fig. 1) and then pathways were dropped in a systematic fashion to create alternate models for comparison, revealing the most parsimonious model (Supplementary material). Models were fitted to raw data using full information maximum likelihood to account for data missing at random and alternative models were compared using chi-square likelihood ratio test of comparative model fit, comparative fit index (CFI), and root mean square error of approximation (RMSEA), along with a number of other parameters (Supplementary material). Structural equation modelling and factor analysis was performed in the statistical modelling software MPLUS version 5 (Muthén and Muthén, 2012).

#### Voxel-based analysis of diffusion images

To identify which regions of white matter may be specifically associated with either apathy or depression, statistical analysis was performed using the randomize program in FSL (Winkler *et al.*, 2014) with 10 000 permutations. A general linear model was used to test positive and negative linear associations between fractional anisotropy/mean diffusivity and (i) geriatric depression scale-apathy score; and (ii) geriatric depression scale-depression, controlling for global cognitive impairment age and gender (dummy coded) as covariates of no interest in a single model (a single general linear model was constructed for fractional anisotropy and mean diffusivity separately). Multiple comparisons were adjusted for using threshold-free cluster enhancement (TFCE; H = 2, E = 0.5, C = 6) and corrected *P*-values were thresholded at *P* ≤ 0.01 and their spatial distribution compared with standard white matter atlases (Harvard-Oxford Cortical and Subcortical Atlas and Johns Hopkins University DTI-based white-matter atlases) (Mori *et al.*, 2005; Hua *et al.*, 2008)*.*

## Results

### Rates of depression and apathy in patients with small vessel disease and controls

Demographic characteristics of the patients with SVD and control populations are shown in Table 1. The cases with SVD had higher apathy (0–6) and depression (0–24) scores than the controls; apathy mean [standard deviation (SD)] 2.7 (1.3) versus; 1.5 (1.5); *P* ≤ 0.001; depression mean (SD) 10.7 (3.1) versus 3.6 (3.4) *P* ≤ 0.001. Using a median cut-off of 3 for the apathy scale, and 10 for depression, 62/120 (52%) of participants with SVD had high apathy and 67/120 (56%) had high depression. There was considerable dissociation between apathy and depression, with 41/120 (34%) of the patients with SVD reporting co-occurring apathy and depression, but with an additional 47/120 (39%) experiencing either apathy or depression in isolation.

### Confirmatory factor analysis of the Geriatric Depression Scale

The confirmatory factor analysis was conducted in MPLUS. The literature has suggested a number of different factor structures for the Geriatric Depression Scale, with four factors being most common, with apathy/social withdrawal consistently occurring as a single factor (Kim *et al.*, 2012). Our results suggested a six factor structure, consistent with that found by Adams and colleagues (2004), with adequate model fit statistics [χ*^2^* (58) = 75, CFI = 0.96 RMSEA = 0.05]. Therefore, both depression (0–24) and apathy variables (0–6) were included in the structural equation model, as described in the ‘Materials and methods’ section.

### Structural equation model of the relationships between white matter microstructure and clinical measures in SVD

Our initial model had adequate model fit statistics [χ^2^(2) = 3.8, *P* = 0.15; CFI = 0.99; RMSEA = 0.08, 90% confidence interval (CI) = 0.0–0.22] and found that while reduction in white matter microstructure (median fractional anisotropy) was a significant predictor of greater apathy scores (standardized coefficient = −0.38, *P* ≤ 0.001) and more cognitive impairment (standardized coefficient = 0.45, *P* ≤ 0.001), it was not significantly related to symptoms of depression (standardized coefficient = −0.16, *P* = 0.07). There was also a significant direct effect of median fractional anisotropy on quality of life (standardized coefficient = 0.23, *P* = 0.01). The model also revealed that apathy (standardized coefficient = −0.22, *P* = 0.01) and depression (standardized coefficient = −0.39, *P* ≤ 0.001) both independently predicted a poorer quality of life, while cognitive impairment (standardized coefficient = 0.12, *P* = 0.19) did not. Apathy, depression and cognitive impairment were all significantly correlated with each other (all *P* ≤ 0.05).

**Figure 2:**
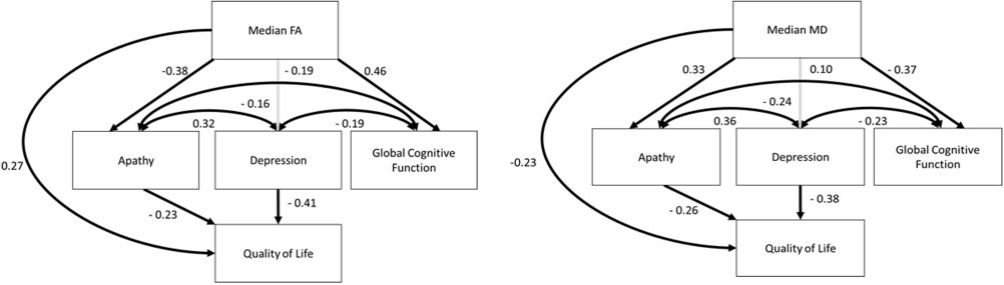
**Final model depicting significant pathways between median fractional anisotropy/median diffusivity and apathy, global cognition and quality of life in patients with SVD.** FA = fractional anisotropy; MD = median diffusivity. NART errors and age are regressed onto all independent variables. Dark lines represent significant paths (*P* ≤ 0.05), while grey lines are non-significant paths. Note: the non-significant path between Global Cognitive Impairment and Quality of Life was dropped to acquire best model fit. The values presented are standardized β coefficients.

We then constrained non-significant pathways to a value of zero in order to test a number of alternative nested models (Supplementary material). This resulted in a series of models with adequate model fit (Supplementary material) but with Model 3 having fit indices comparable to our hypothesized model, to which it was identical, but with the non-significant path from cognitive impairment to quality of life being constrained to zero [χ^2^(3) = 5.4, *P* = 0.15; CFI = 0.98; RMSEA = 0.08, 90% CI = 0.0–0.22; Fig. 2]. A χ^2^ difference test revealed no significant difference between the hypothesized model and Model 3 [χ^2^(1) = 1.6, *P* = 0.21], and therefore Model 3 was accepted as the most parsimonious.

We repeated the final model substituting the median fractional anisotropy value with median mean diffusivity, and found consistent results [χ^2^(2) = 3.6, *P* = 0.16; CFI = 0.99; RMSEA = 0.08, 90% CI = 0.0–0.22]. Again we found that apathy (standardized coefficient = 0.33, *P* ≤ 0.001) and cognitive functioning (standardized coefficient = −0.37, *P* ≤ 0.001), but not depression (standardized coefficient = 0.10, *P* = 0.52; Fig. 2), were significantly associated with median mean diffusivity (Fig. 2). The correlations between all the key variables included in the models can be found in Table 2.

**Table 2:** Pearson’s correlations between key variables included in the structural equation model

	Median FA	Median MD	Depression	Apathy	Cognitive function	Processing speed	Executive function	Memory	Quality of life	Age
Median FA	−									
Median MD	−0.90**	−								
Depression (GDS 0–24)	−0.12	−0.02	−							
Apathy (GDS 0–6)	−0.38**	−0.34**	0.51**	−						
Cognitive function	0.43**	−0.37**	−0.23*	−0.32**	−					
Processing speed	0.23**	−0.34**	−0.10	−0.20*	0.85**	−				
Executive function	0.23**	−0.16^#^	−0.02	−0.01	0.28**	0.23*	−			
Memory	0.15	−0.10	−0.10	−0.10	0.18*	0.18*	0.68**	−		
Quality of life	0.41**	−0.38	−0.65**	−0.58**	0.35**	0.26*	0.11	−0.01	−	
Age	−0.06	0.01	−0.12	0.14	−0.09	−0.10	−0.18*	−0.10	−0.05	−
NART	0.21*	−0.15	−0.14	−0.06	0.66**	0.45**	0.10	0.13	0.03	−0.14

GDS = Geriatric Depression Scale; FA = fractional anisotropy; MD = median diffusivity; NART = National Adult Reading Test; Cognitive function = global cognitive composite score; ^#^ *P* ≤ 0.10; * *P* ≤ 0.05; ** *P* ≤ 0.01.

### Structural equation models of the effect of cognitive domains on the relationship between white matter and apathy

**Figure 3:**
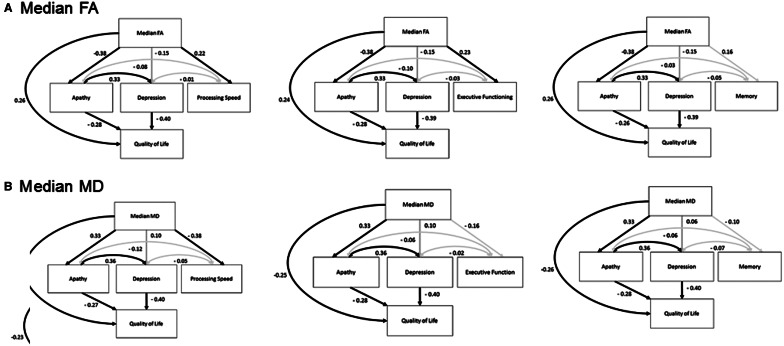
***Post hoc* structural equation model investigating the possible mediating effect of executive functioning and memory on the relationship between apathy and median fractional anisotropy.** FA = fractional anisotropy; MD = median diffusivity. NART errors and age are regressed onto all independent variables. Dark lines represent significant paths (*P* ≤ 0.05), while grey lines are non-significant paths. Note: the non-significant path between Global Cognitive Impairment and Quality of life was dropped to acquire best model fit. The values presented are standardized β coefficients.

To ensure the relationships between white matter changes, apathy and depression were not mediated by specific cognitive domains we conducted a *post hoc* analysis using executive functioning, memory and processing speed performance instead of global cognitive functioning. The executive function [χ^2^ (2) = 2.9, *P* = 0.23; CFI = 0.99; RMSEA = 0.06, 90% CI = 0.0–0.20], long-term memory [χ^2^(2) = 2.7, *P* = 0.25; CFI = 0.99; RMSEA = 0.06, 90% CI = 0.0–0.20] and processing speed models [χ^2^(2) = 2.8, *P* = 0.25; CFI = 0.99; RMSEA = 0.06, 90% CI = 0.0–0.20] all had adequate model fit statistics, and reliably showed that regardless of the cognitive measure used, a reduced median fractional anisotropy significantly predicts more apathy, poorer cognitive function and a worse quality of life, but that depression was not significantly related to white matter changes (see Fig. 3 for full models). These models are consistent with the previous analyses, confirming that the relationship between white matter changes and apathy is maintained when accounting individually for each of the following cognitive domains: executive functioning, memory and processing speed. These models were repeated using the median mean diffusivity value and showed consistent results (Fig. 3).

### White matter correlates of apathy and depression

**Figure 4:**
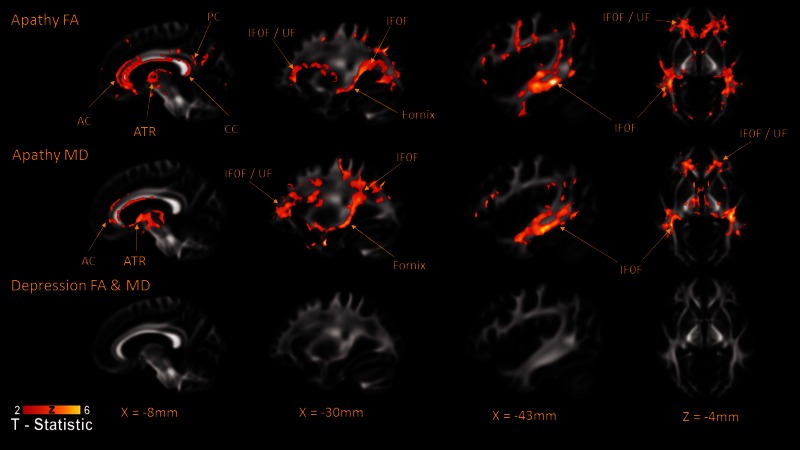
**Areas of reduced fractional anisotropy/median diffusivity associated with apathy in patients with SVD, controlling for age gender and cognitive functioning.** Images displayed using neurological convention. AC = anterior cingulum; ATR = Anterior Thalamic Radiation; CC = corpus callosum; FA = fractional anisotropy; IFOF = inferior fronto-occipital fasciculus; MD = mean diffusivity; PC = posterior cingulum; UF = uncinate fasciculus.

Consistent with the structural equation model, when controlling for depression, apathy was found to be significantly related to widespread changes in both fractional anisotropy and mean diffusivity (Fig. 4). Fractional anisotropy and mean diffusivity associations with apathy were primarily in anterior brain regions, but also areas within the parietal and temporal lobes. This included significant results within the bilateral anterior cingulum, corpus callosum, fornix, uncinate/inferior fronto-occipital fasciculus and the anterior thalamic radiation, including portions of the anterior limbs of the external capsule. In contrast, when controlling for apathy, we found no significant relationship between our white matter parameters and symptoms of depression. The analysis was also repeated using the whole Geriatric Depression Scale and again there were no significant findings. These findings strongly suggest the involvement of limbic-cortical-thalamic-striatal circuits in apathy in people with SVD. As the regions associated with apathy in this analysis have previously been associated with processing speed (Tuladhar *et al.*, 2015), we repeated the analysis specifically controlling for this cognitive domain, finding consistent results (Supplementary material).

## Discussion

In this study, we first determined the presence of apathy and depression in SVD compared to a large sample of healthy controls, and investigated the overlap between apathy and depression in SVD, before determining the relationship between white matter microstructure and apathy, depression and quality of life. Finally, we investigated regional associations between white matter microstructure and apathy to identify possible mechanisms that may underlie these symptoms.

These results demonstrate that both apathy and depression are increased in patients with SVD, but that frequently they do not occur together in the same patient. We found that while 34% of patients with SVD displayed both symptoms concurrently, 39% had dissociated symptoms of either depression or apathy. Of particular significance is our finding that ∼18% who are not depressed had high levels of apathy as this may frequently be missed in the clinical setting.

Despite evidence that apathy is a common neuropsychiatric symptom following stroke (Hackett *et al.*, 2014), there are little data on its impact on patients’ quality of life. Previous studies in SVD have reported that both cognitive functioning (Brookes *et al.*, 2014*b*) and depression (Brookes *et al.*, 2013) following stroke are associated with a poorer quality of life. Here we demonstrate that both apathy and depression each have their own independent contributions to the quality of life in SVD. In our model, cognitive impairment impacted indirectly on quality of life through its association with apathy and depression. This serves to highlight the importance of the assessment of emotional neuropsychiatric symptoms such as apathy and depression in patients with SVD. We also found a direct path between white matter damage and quality of life. This effect is most likely mediated by one or more factors not included in the modelling because of model size constraints, for example, physical disability or motor problems.

It has been suggested that the increased prevalence of apathy and depression in SVD may be caused by white matter damage to the cortico-subcortical pathways that connect brain regions important for regulating emotion (Taylor *et al.*, 2013). DTI of these patients revealed that white matter microstructural changes in SVD are associated with increased apathy, but not depressive symptoms. Spatial analysis identified a number of subcortical regions commonly thought to be affected by SVD pathology as underlying the association between white matter changes and apathy (Prins *et al.*, 2005). This included the anterior cingulum, fornix, uncinate fasciculus, inferior frontal occipital fasciculus and the body and genu of the corpus callosum.

### The anterior cingulate cortex, goal-directed behaviour and apathy

The cingulum is a large white matter tract underlying the cingulate cortex (Shackman *et al.*, 2011), which has been linked to both affect regulation and cognitive control, and has been associated with apathy in both structural (Apostolova *et al.*, 2007; Stanton *et al.*, 2013) and functional MRI studies (Marshall *et al.*, 2007; Alexopoulos *et al.*, 2013). Consistent with our current findings, reductions in cingulum white matter integrity have also been associated with apathy in patients with dementia and in normal ageing (Cacciari *et al.*, 2010; Kim *et al.*, 2011; Hahn *et al.*, 2013; Spalletta *et al.*, 2013). In particular, lesions, including strokes, involving the anterior cingulate cortex can lead to profound apathy often referred to as abulia (Cohen *et al.*, 1999; Grunsfeld and Login, 2006). It has now been suggested that in conjunction with the ventral striatal reward systems and frontal regions important for task initiation, the anterior cingulate cortex has a role in the selection and maintenance task performance (Holroyd and Yeung, 2012). Given that, in addition to the cingulum, we also found significant associations between apathy and areas of both the internal and external capsules and diffuse regions of frontal white matter, one hypothesis is that apathy in SVD is caused by disruption of the connections between frontal-striatal circuits, including the anterior cingulate cortex, which select and initiate goal-directed behaviour based on previous reward information.

### The fornix, episodic memory and apathy

There was a significant association between apathy and white matter structural changes to the fornix and the anterior thalamic radiation. Reduction in fornix white matter integrity has typically been associated with episodic memory (Metzler-Baddeley *et al.*, 2012). This suggests that an ability to access autobiographical information about times, places and their associated emotions or reward value may be important in apathy. However, our structural equation model revealed that performance on memory tasks was not related to apathy scores. It is possible that the relationship between apathy and memory may be isolated to only affective or reward related memories, and future studies may be able to investigate this using experimental paradigms rather than more generic neuropsychological tests.

### The uncinate fasciculus, mnemonic associations and apathy

Consistent with studies in Alzheimer’s disease (Hahn *et al.*, 2013), progressive supranuclear palsy (Agosta *et al.*, 2014) and normal ageing (Grool *et al.*, 2014), we found a significant relationship between apathy and the uncinate fasciculus. The uncinate fasciculus is considered to be important for a range of cognitive functions such as episodic memory (Metzler-Baddeley *et al.*, 2012), linguistic ability (Papagno *et al.*, 2011) and social-emotional ability (Hornberger *et al.*, 2011). It has recently been demonstrated that apathy in those with late-life depression is significantly associated with fractional anisotropy changes in the left uncinate fasciculus and that reduced structural integrity in this region may mediate treatment response (Yuen *et al.*, 2014).

There was also a significant association between apathy and the inferior fronto-occipital fasciculus, both at its anterior termination in the frontal operculum, but also more posteriorly (Fig. 3). The inferior fronto-occipital fasciculus projects from the frontal operculum and proceeds posteriorly passing though the insular to posterior temporal lobe and finally the occipital lobe (Martino *et al.*, 2010). There is little evidence for a direct role for the inferior fronto-occipital fasciculus in apathy. However, Stanton and colleagues (2013) reported that a reduced insula volume, as well as atrophy in the cingulate region, was associated with high levels of apathy in patients with neurodegenerative disorders.

### Apathy or depression in small vessel disease?

Given that our results are not consistent with the previous finding in SVD that the white matter tracts implicated here have a role in depressive symptoms, it is essential to discuss why this may be. First, it is important to state that based on this evidence alone, we are not suggesting that people with SVD do not suffer from symptoms of depression. Rather, these findings suggest that depression in SVD may to some extent be secondary to motivational loss and cognitive impairment, which are the more direct consequence of white matter damage. Second, while our results indicate that there is no direct association between white matter integrity and depression, our data do suggest that depression still has a significant impact on functional outcomes in patients with SVD.

Our results show a high level of correspondence with the only other paper that has investigated regional associations between white matter and depression in SVD (van Uden *et al.*, 2015). In that study, depressive symptoms were measured using the Centre for Epidemiologic Studies Depression Scale (CES-D; Radloff, 1977). The CES-D does contain some questions that tap into apathy, including questions such as ‘I could not get going’ and ‘I talked less than normal’, which may have driven the significant associations between depression and white matter microstructure in this study.

### Conclusions, limitations and future directions

Based on the results of this study we concluded that apathy is a significant symptom in people with SVD and is associated with damage to white matter tracts which connect regions in the frontal lobe with both subcortical structures and the temporal lobe. Taking evidence from a wide range of literature we have inferred that apathy may be caused by damage to a number of networks responsible for the integration of reward, mnemonic associations and the selection and initiation of goal-directed behaviour. Future research should now focus on probing these regions with behavioural and cognitive tasks designed to elicit reward-related behaviour in combination with functional MRI. Adam and colleagues (2013) have shown that a reward-based decision-making task is sensitive to treatment of profound apathy with a dopamine agonist in a patient with bilateral basal–ganglia lesions as a result of ischaemic stroke. While Kohno *et al.* (2010) demonstrated the potential benefit of Ropinirole, another dopamine receptor agonist, to reduce apathy in a patient who had suffered an ischaemic lesion to the prefrontal cortex.

This is the first study to show specific relationships between white matter pathology and apathy in people with SVD. We have also supported this by using structural equation modelling to demonstrate that both apathy and depression can be dissociated in SVD and have independent associations with quality of life. However, future research would benefit from taking a more detailed approach when assessing apathy. For instance, by using the Lille Apathy Rating Scale (Sockeel *et al.*, 2006), which divides apathy into subdomains enabling the development of patient specific profiles, also determining whether the current results can be replicated. Furthermore, although we carefully selected our voxel-based analysis methodology (Avants *et al.*, 2011) to reduce common problems with voxel-based methods (Ashburner and Friston, 2000; Bookstein, 2001), the localization of apathy within the white matter should be interpreted with caution and replicated in an independent sample of patients with SVD.

It is important to note that despite our findings that apathy is significantly related to white matter microstructure in SVD, even when controlling for cognitive functioning, some regions do overlap with those reported to be associated with cognitive functioning. This is particularly the case for processing speed, which has also been shown to be related to diffuse white matter changes particularly in frontal regions (Tuladhar *et al.*, 2015).

This study has a number of clinical implications. Firstly, our findings that apathy and depression are dissociable in SVD, and that they both impact significantly on patient quality of life, suggests a more routine assessment of apathy is warranted. Secondly, an ability to accurately distinguish between apathy and depression, whether they occur together or in isolation from each other, may allow the development of distinct treatment approaches for each. For instance, while antidepressant treatments such as serotonin re-uptake inhibitors (SSRIs) may be effective for symptoms of depression (Salzman *et al.*, 2002), they may not be effective at treating apathy and its specific contribution to a person’s functional outcome. While there is limited literature on effective treatments for apathy, a number of dopaminergic compounds have been suggested (Kohno *et al.*, 2010; Rosenberg *et al.*, 2013; Thobois *et al.*, 2013). In addition, evidence suggests that the presence of symptoms of apathy reduces the effectiveness of treatments for depression (Chaturvedi and Sarmukaddam, 1986; Hölttä *et al.*, 2012). It is currently unclear whether this is only due to a reduced engagement with the treatment regime or if neurobiological differences between those who have both symptoms of apathy and depression versus those with depression alone may play a part. A clear delineation of divergent properties of apathy and depression, both at the behavioural level and the neurobiological level, may allow the development of treatment plans for apathy itself or treatments for post-stroke depression that take into account the presence or absence of apathy.

## Funding

The SCANS study was supported by a Wellcome Trust grant (081589). Recruitment to the SCANS study was supported by the English National Institute of Health Research Clinical Stroke Research Network. Matthew Hollocks is supported by a Stroke Association/British Heart Foundation Project Grant (TSA/BHF Prog 2010/01). Andrew Lawrence is supported by an Alzheimer’s Research UK project grant (ARUK-PG2013-2). Masud Husain is supported by a Wellcome Trust Principal Research Fellowship. Hugh Markus is supported by an NIHR Senior Investigator award and by the Cambridge University Hospitals Department of Health’s NIHR Comprehensive Biomedical Research Centre.

## Supplementary material

Supplementary material is available at *Brain* online.

Supplementary material

## Abbreviations

CFI: comparative fit index
RMSEA: root mean square error of approximation
SVD: small vessel disease
