# Supplementary material for: Differential relationships between apathy and depression with white matter microstructural changes and functional outcomes
Source: Brain. 2015 Oct 21;138(12):3803–15. doi: 10.1093/brain/awv304 (PMC4655344; doi:10.1093/brain/awv304)
Supplement: Supplementary material [file brain_awv304_index.html]

Supplementary Data | Brain

## Supplementary Data

files

- Supplementary Data - pdf file
- Supplementary Data - pdf file
- Supplementary Data - pdf file
- Supplementary Data - jpg file
