# Supplementary material for: Differential relationships between apathy and depression with white matter microstructural changes and functional outcomes
Source: Brain. 2015 Oct 21;138(12):3803–15. doi: 10.1093/brain/awv304 (PMC4655344; doi:10.1093/brain/awv304)
Supplement: Supplementary material [file 09c4924e46338747fc885566dd280cab_brain-2015-00973-File008.pdf]

Supplementary Materials

Supplement 1

Cognitive indices and task measures

Cognitive Index	Task & Normative Data reference	Task Measure(s) Used & Additional Details
Executive Function (EF)		
		[1] Time to complete Part B (number-letter switching)
	Verbal Fluency [2]	Total number of Correct Words generated
	Modified Wisconsin Card Sort Test [3]	Categories Achieved & Perseverative Errors*
Processing Speed (PS)		
	BMIPB Speed of Information Processing [4]	Total correct, adjusted for motor score & errors (%)*
	Digit Symbol Substitution [5]	Total Correct
	Grooved Pegboard Task [6]	Time to complete (average of 2 hands)
Working Memory (WM)		
	Digit Span Task [5]	Total Score
Long Term (Episodic) Memory (LTM)		
	WMS-III Logical Memory [7]	Total Score: Immediate Recall & Delayed Recall*
	WMS-III Visual Reproduction [7]	Total Score: Immediate Recall & Delayed Recall*
Performance Intelligence (PIQ)		
	WASI Block Design [8]	Total Score
	WASI Matrix Reasoning [8]	Total Score
Verbal Intelligence (VIQ)		
	WASI Vocabulary [8]	Total Score
	WASI Similarities [8]	Total Score

*BMIPB - Birt Memory & Information Processing Battery; WMS-III - Wechsler Memory Scale - Third Edition (UK); WASI - Wechsler Abbreviated Scale of Intelligence. *Composite score used for multiple task measures.*

[1] Mitrushina M, Boone KB, Razani J, D'Elia LF (2005) Handbook of Normative Data for Neuropsychological Assessment. USA: Oxford University Press, second edition.

[2] Delis DC, Kaplan E, Kramer JH (2001) Delis-Kaplan Executive Function Scale (D-KEFS). San Antonio, TX: The Psychological Corporation.

[3] Nagahama Y, Okina T, Suzuki N, Matsuzaki S, Yamauchi H, et al. (2003) Factor structure of a modified version of the wisconsin card sorting test: an analysis of executive deficit in Alzheimer's disease and mild cognitive impairment. Dementia and Geriatric Cognitive Disorders 16: 103–112.

[4] Coughlan AK, Oddy M, Crawford JR (2007) The BIRT Memory and Information Processing Battery (B-MIPB). Wakefield, UK: The Brain Injury Rehabilitation Trust (BIRT).

[5] Wechsler D (1997) Wechsler Adult Intelligence Scale-Third edition (WAIS-III). San Antonio, TX: The Psychological Corporation.

[6] Dawson JD, Uc EY, Anderson SW, Johnson AM, Rizzo M (2010) Neuropsychological predictors of driving errors in older adults. Journal of the American Geriatrics Society 58: 1090–1096.

[7] Wechsler D (1997) Wechsler Memory Scale - Third Edition (WMS-III UK) Administration and Scoring Manual. San Antonio, TX: The Psychological Corporation.

[8] Wechsler D (1999) Wechsler Abbreviated Scale of Intelligence (WASI) Manual. San Antonio, TX: The Psychological Corporation.

Cerebral small vessel disease: from pathogenesis and clinical characteristics to therapeutic
