# Supplementary material for: Differential relationships between apathy and depression with white matter microstructural changes and functional outcomes
Source: Brain. 2015 Oct 21;138(12):3803–15. doi: 10.1093/brain/awv304 (PMC4655344; doi:10.1093/brain/awv304)
Supplement: Supplementary material [file dd003657587499e5e60d71cdaaab0b6f_brain-2015-00973-File009.pdf]

Supplement 2. Table comparing model fit indices for the original hypothesised model (Model 1) and alternative nested models

**Model**		***x^2 (d.f.)***	**CFI**	**TLI**	**RMSEA**
**Model 1**	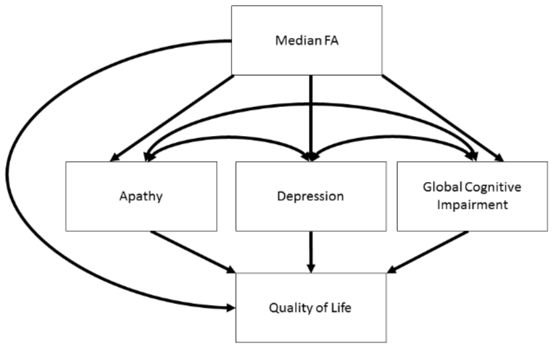	3.8 (2)	0.986	0.855	0.085
**Model 2**	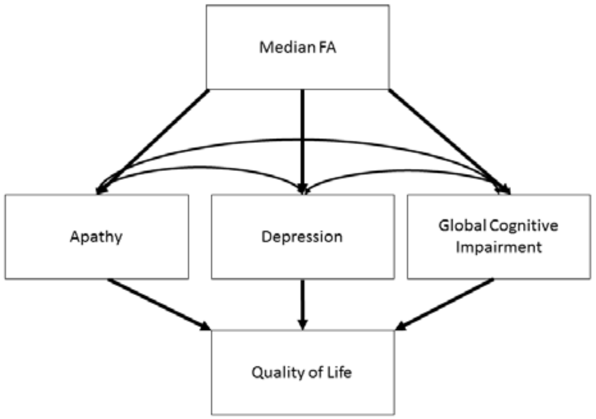	9.5 (3)	0.947	0.648	0.113

**Model 3**	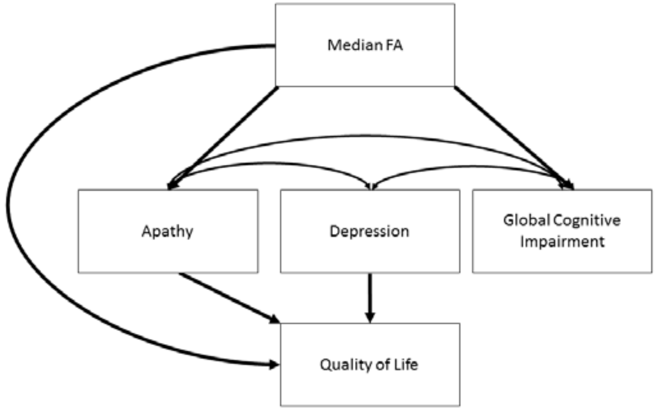	5.4 (3)	0.981	0.870	0.081
**Model 4**	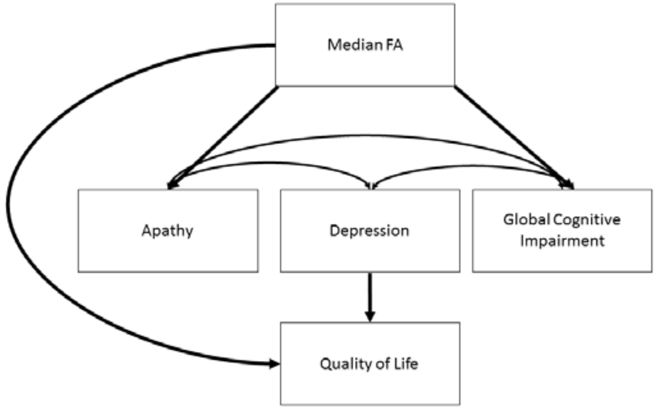	12.2 (4)	0.933	0.666	0.129

**Model 5**	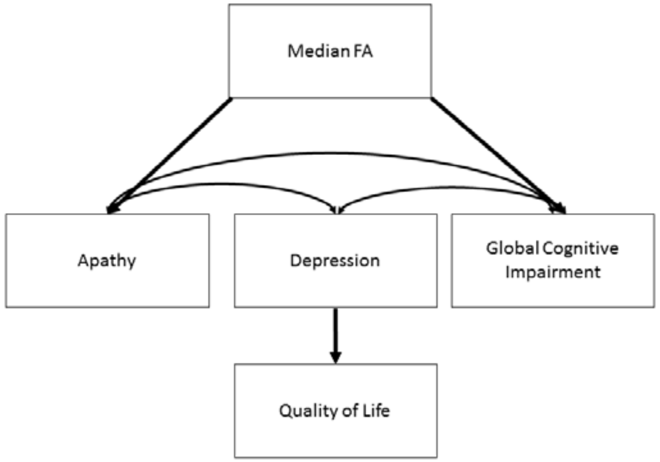	28.5 (5)	0.810	0.242	0.195

CFI = Comparative Fit Index, TLI = Tucker Lewis Index, RMSEA = Root Mean Square Error of Approximation
