# Supplementary material for: Differential relationships between apathy and depression with white matter microstructural changes and functional outcomes
Source: Brain. 2015 Oct 21;138(12):3803–15. doi: 10.1093/brain/awv304 (PMC4655344; doi:10.1093/brain/awv304)

Supplement 3. Areas of increased FA/MD associated with apathy in patients with small vessel disease, controlling for age gender and processing speed.

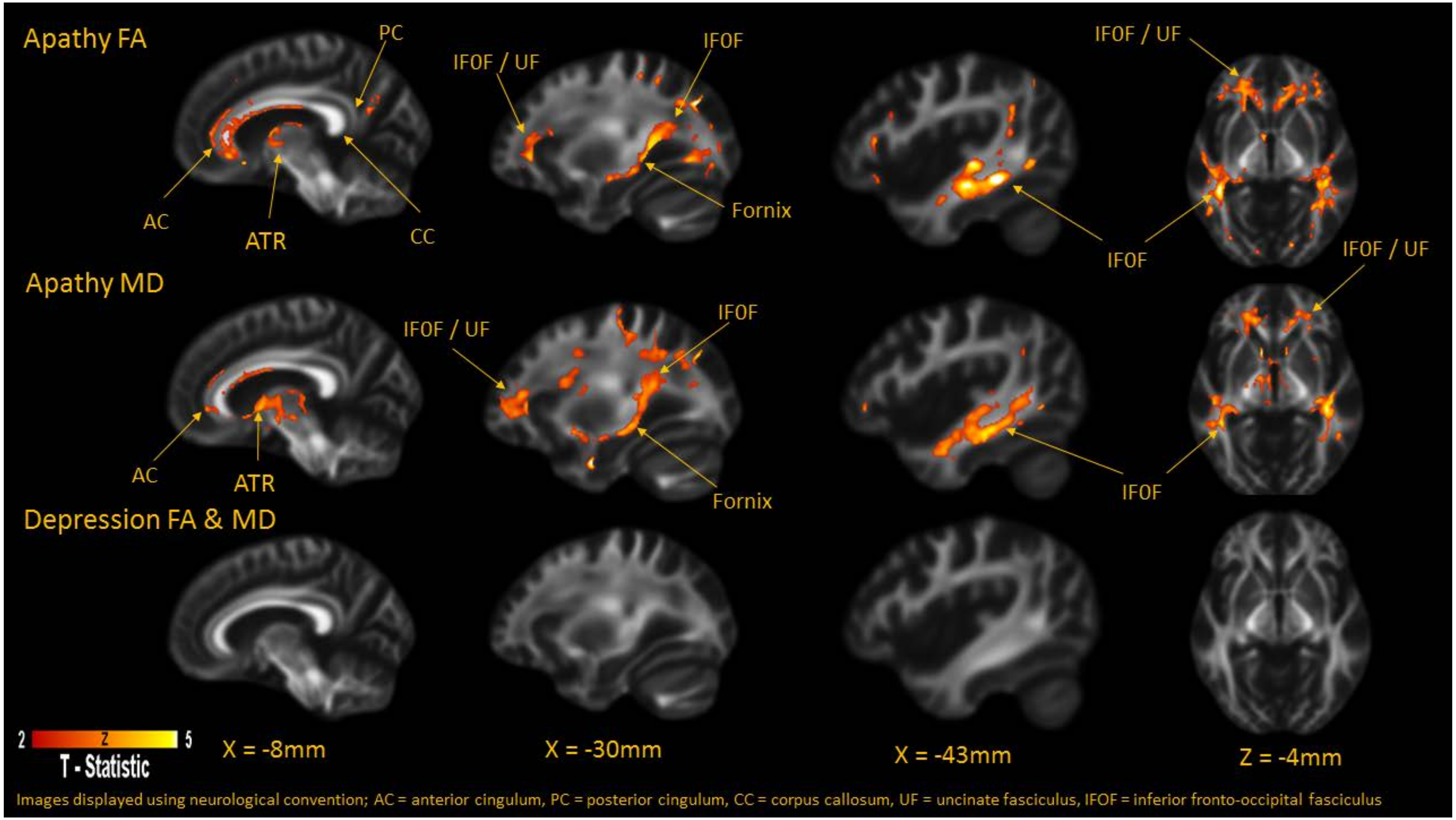

Supplement: Supplementary material [file 76e15b7ebd1cdb07b564523244396751_brain-2015-00973-File010.pdf]
