# Supplementary figures and images for: Differential relationships between apathy and depression with white matter microstructural changes and functional outcomes
Source: Brain. 2015 Oct 21;138(12):3803–15. doi: 10.1093/brain/awv304 (PMC4655344; doi:10.1093/brain/awv304)

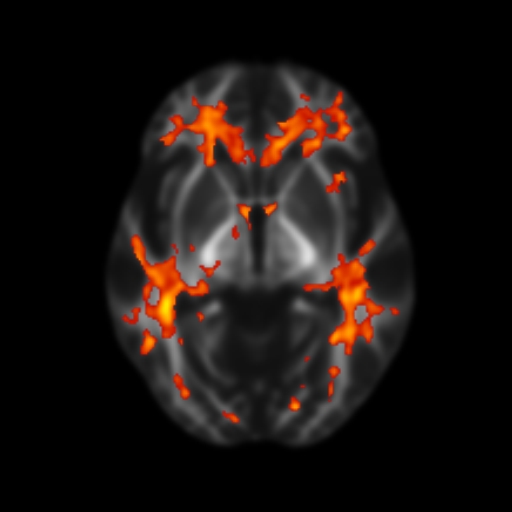

Supplement: Supplementary material [file 045fbcea7bfd0f39ac73858dcad17a4b_brain-2015-00973-File011.jpg]
